# Duration of Dual Antiplatelet Therapy in Patients With Acute Coronary Syndrome Treated With New Generation Stents: A Meta-Analysis of Randomized Controlled Trials

**DOI:** 10.3389/fcvm.2021.615396

**Published:** 2021-02-03

**Authors:** Wen-Jiao Zhang, Xuan Qiao, Wen-Fen Guo, Xi-Ying Liang, Yan Li, Zhi-Lu Wang

**Affiliations:** ^1^The First Clinical Medical College of Lanzhou University, Lanzhou, China; ^2^Department of Cardiology, Baiyin Third People's Hospital, Baiyin, China; ^3^Department of Cardiology, The First Hospital of Lanzhou University, Lanzhou, China

**Keywords:** duration, dual antiplatelet therapy, new generation stent, percutaneous coronary intervention, acute coronary syndrome

## Abstract

**Background and Objective:** The optimum duration of dual antiplatelet therapy (DAPT) remains uncertain in patients with acute coronary syndrome treated with new generation stents. This meta-analysis was performed to investigate ischemia and bleeding outcomes with different DAPT strategies.

**Methods:** PubMed, Embase, Cochrane and Web of science from inception to May 27, 2020, were systematically searched. Randomized controlled trials were included to compare short-term (6 months or less) with standard (12 months) DAPT in patients with acute coronary syndrome treated with new generation stents. The primary endpoints were myocardial infarction, definite or probable stent thrombosis and major bleeding. The secondary endpoints included all-cause death, cardiovascular death, stroke, target vessel revascularization and net adverse clinical events. Random effect model and fixed effect model were used to calculate the odds ratio (OR) and 95% confidence interval (CI) of each endpoint.

**Results:** Four randomized controlled trials and seven subgroup analyses of larger randomized controlled trials, including a total of 21,344 patients with acute coronary syndrome, met our inclusion criteria. The shorter DAPT was associated with significantly lower major bleeding compared with the standard DAPT (OR 0.71, 95% CI 0.56–0.90, *P* = 0.005, *I*^2^ = 25%), while without increasing the risk of myocardial infarction (OR 1.18, 0.88–1.58, *P* = 0.28, *I*^2^ = 20%), definite or probable stent thrombosis (OR 1.60, 0.98–2.59, *P* = 0.06, *I*^2^ = 0%). No significantly difference was observed in the risk of all-cause death (OR 0.96, 0.72–1.27, *P* = 0.76, *I*^2^ = 2%), cardiovascular death (OR 0.91, 0.62–1.33, *P* = 0.62, *I*^2^ = 0%), stroke (OR 0.84, 0.54–1.30, *P* = 0.43, *I*^2^ = 0%), target vessel revascularization (OR 1.14, 0.84–1.55, *P* = 0.41, *I*^2^ = 8%), and net adverse clinical events (OR 0.93, 0.80–1.07, *P* = 0.3, *I*^2^ = 18%) between the two groups.

**Conclusions:** In patients with acute coronary syndrome treated with new generation stents, the shorter DAPT leads to a marked reduction in the risk of major bleeding compared with the standard DAPT. This benefit is achieved without increasing the risk of mortality or ischemic outcomes. The study protocol was registered in PROSPERO (CRD42020189871).

## Introduction

Dual antiplatelet therapy (DAPT) of aspirin and P2Y_12_ inhibitors has been shown to reduce stent thrombosis after drug-eluting stents (DES). Based on PCI-CURE clinical trial (1), a standard DAPT regimen for 12 months was recommended by acute coronary syndrome guidelines (2, 3). With the modification of the antiproliferative drug, the stent polymer and the stent platform in new generation of DES, the thrombosis of late and extremely advanced stents decreased significantly compared with first-generation of DES (4). The studies of second-and third-generation of DES by optical coherence tomography confirmed that neointimal coverage could be completed within 3–6 months, which suggested that it was feasible to shorten the duration of DAPT (5, 6). Prolonged the duration of DAPT increases the risk of bleeding, is costly, and can delay selective and semi-selective surgery. Meanwhile, with more effective antiplatelet agent, avoiding bleeding may be more important than minimize the risk of ischemic events by prolonging the duration of DAPT.

In patients with acute coronary syndrome, increased platelet activation and aggregation play a central role, and adequate platelet inhibition is essential to reduce the risk of recurrent ischemic events, including stent thrombosis and myocardial infarction. Several larger randomized controlled trials have reported comparable incidences of recurrent ischemic events in patients with acute coronary syndrome treated with short-term and standard DAPT (7, 8). However, other studies have shown that shortening the duration of DAPT after percutaneous coronary intervention (PCI) may increase the risk of stent thrombosis and myocardial infarction in patients with acute coronary syndrome compared with standard DAPT (9, 10). Therefore, the optimal duration of DAPT in patients with acute coronary syndrome followed DES has always been a topic of debate and research.

In recent years, many large-scale clinical trials have explored safety and efficacy of shortening the duration of DAPT after implanted with new generation of DES in patients with acute coronary syndrome (8, 10). However, these analyses do not have sufficient evidence to detect differences for relatively rare events such as myocardial infarction or definite or probable stent thrombosis. Therefore, a hypothesis has been proposed that shortening the duration of DAPT can preserve ischemic protection and may reduce bleeding complications. In this context, a meta-analysis was conducted to determine the safety and efficacy of short-term (≤6 months) DAPT compared with standard 12 months DAPT in patients with acute coronary syndrome implanted with new generation stents.

## Methods

### Literature Search and Selection Criteria

PubMed, Embase, Cochrane and Web of Science database were searched from their earliest records until May 27, 2020. All relevant combinations of following keywords “acute coronary syndrome,” “percutaneous coronary intervention,” “drug-eluting stents,” “platelet aggregation inhibitors,” “aspirin,” “prasugrel,” “ticagrelor,” “clopidogrel,” and “dual antiplatelet therapy” were included for database search without language restrictions. An update reminder for PubMed was created to keep up with the latest research. The inclusion and exclusion criterion of the study met the following requirements: (1) randomized controlled trial (or subgroup analysis of a randomized controlled trial) that compared short-term DAPT (≤6 months) with standard DAPT (12 months) in patients with acute coronary syndrome undergoing PCI; (2) more than 90% of patients were implanted with new generation stents and (3) more than 10% of patients with first generation of DES or bare metal stents were excluded. The new generation stent is defined as any DES after the first-generation of DES. Title, abstract, and full text were screened independently to determine the study that met the inclusion and exclusion criteria by two investigators (Zhang WJ and Qiao X). Conflicts between reviewers were resolved by consensus. The risk of bias for each randomized controlled trial was assessed using the Cochrane Collaboration's tool (11). Grading of Recommendations Assessment, Development and Evaluation (GRADE) was used to assess the quality of evidence (12). Because these analyses were based on previously published studies, there was no requirement for ethical approval and patient consent. The study protocol was registered in PROSPERO (CRD42020189871).

### Data Extraction and Definitions of Endpoints

Data extraction and analysis were conducted in adherence to the Preferred Reporting Items for Systematic Reviews and Meta-analysis (PRISMA) statement (13), and intention-to-treat analysis was employed. The baseline characteristics of patients and trials were extracted by two researchers independently, and the divergences were resolved through negotiation (Wang ZL). The primary endpoints were consisted of myocardial infarction, definite or probable stent thrombosis and major bleeding. The secondary endpoints included all-cause death, cardiovascular death, stroke and target-vessel revascularization, and net adverse clinical events. Major bleeding was defined per trial definition or Bleeding Academic Research Consortium (BARC) ≥ 3 (14). Net adverse clinical events were defined as applied in each trial or combination of ischemia and bleeding events of individual publication.

### Statistical Analysis

Odds ratio (OR) and 95% confidence intervals (95% CI) were used as summary statistics. Heterogeneity across trials was assessed by the Cochran Q statistic and the Higgins *I*^2^ test. In case of substantial heterogeneity (*I*^2^ ≥ 50%), random effect model was used, otherwise fixed effect model was applied for calculate the pooled OR. Subgroup analysis was utilized to explore the stability of final results. All tests were two-sided, and a *P* < 0.05 was considered statistically significant. Analyses were conducted using Review Manager Version 5.3 software (The Nordic Cochrane Centre, Copenhagen, Denmark) and Stata version 12.0 software (Statacorp LP, College Station, Texas, USA). The GRADE profiler version 3.6 software was used to evaluate evidence quality and recommendation level. Visual estimation of funnel plot and the Begg's and Egger's tests were performed to investigate the possibility of publication bias.

### Trial Sequential Analysis Performance

The trial sequential analysis was performed to assess the random errors and to calculate the required information size by using an α of 0.05 and a power of 0.8, and the control event proportions were calculated in the meta-analysis.

## Results

### Search Results and Study Characteristics

**Figure 1:**
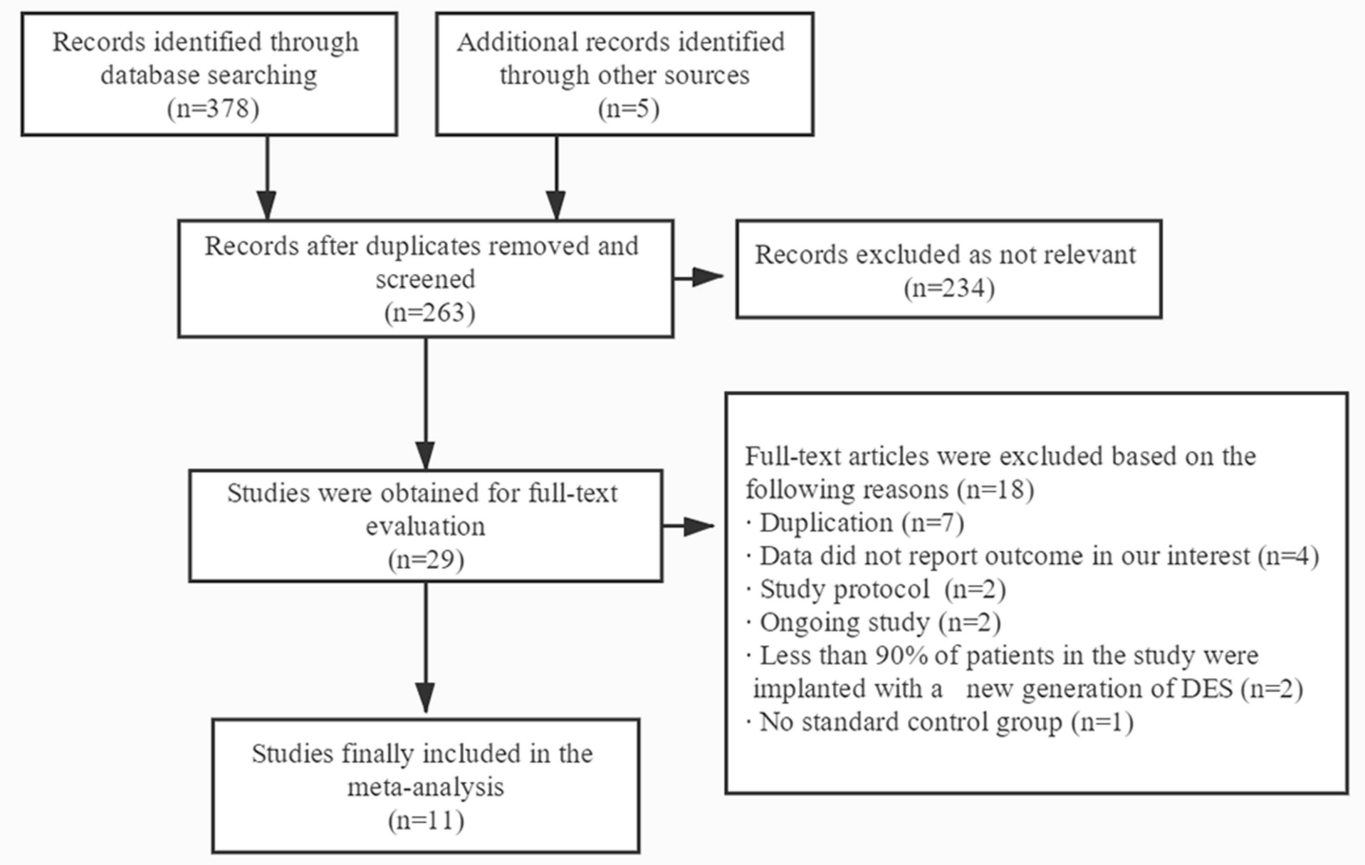
Flow diagram of literature search.

A total of 383 publications were found at initial search, and 29 studies were initially identified (Figure 1). For the following reasons, 18 studies were further excluded: duplication (*n* = 7), data did not report outcome in our interest (*n* = 4), study protocol (*n* = 2), ongoing study (*n* = 2), <90% of patients in the study were implanted with a new generation of DES (*n* = 2) and no standard control group (*n* = 1). Therefore, 11 trials were finally included with a total of 21,344 patients with acute coronary syndrome, including four randomized controlled trials and seven subgroup analyses of larger randomized controlled trials (7–10, 15–21). In the 11 trials, 10,606 patients were divided into short-term (≤6 months) DAPT group and 10,614 patients were divided into standard 12 months DAPT group. The baseline characteristics of the included trials were reported (Table 1). The duration of follow-up in the studies included was from 12 to 24 months. The baseline characteristics of patients included were summarized (Table 2). The average age of the patients included in the study was between 59.8 and 69.1 years old, 74.6% of them were males and 28.1% of them were patients with diabetes mellitus. Antiplatelet agents for the DAPT regimen were aspirin combined clopidogrel (66.3%), ticagrelor (28.5%) or prasugrel (4.3%). Patients with acute coronary syndrome included ST-elevation myocardial infarction (31%), non-ST-elevation myocardial infarction (30.5%) and unstable angina pectoris (33.1%). This meta-analysis included the following new generation stents: the second-generation of DES were consisted of zotarolimus-eluting stent (15.9%) and everolimus-eluting stent (16.7%), and the third-generation of DES included biodegradable polymer sirolimus-eluting stent (28.4%) and biolimus A9-eluting stent (39%).

**Table 1:** Baseline characteristics of the included trials.

**Study name**	**RESET**	**OPTIMIZE**	**ITALIC**	**I-LOVE-IT2**	**IVUS-XPL**	**SMART-DATE**	**DAPT-STEMI**	**GLOBAL LEADERS**	**REDUCE**	**STOPDAPT-2**	**TICO**
Publation year	2012	2013	2014	2016	2016	2018	2018	2018	2019	2019	2020
Study country	Korea	Brazil	Europe and the Middle East	China	Korea	South Korea	Netherlands, Norway, Poland, Switzerland	18 countries	Europe and Asia	Japan	South Korea
Design	RCT	RCT	RCT	RCT	RCT	RCT	RCT	RCT	RCT	RCT	RCT
DAPT strategy	Aspirin + clopidogrel	Aspirin + clopidogrel	Aspirin + clopidogrel	Aspirin + clopidogrel	Aspirin + clopidogrel	Aspirin + clopidogrel/prasugrel/ticagrelor	Aspirin + clopidogrel/prasugrel/ticagrelor	Aspirin + clopidogrel/ticagrelor	Aspirin + clopidogrel/prasugrel/ticagrelor	Aspirin + prasugrel	Aspirin + clopidogrel/prasugrel/ticagrelor
Comparison of DAPT duration (months)	3 vs. 12	3 vs. 12	6 vs. 12	6 vs. 12	6 vs. 12	6 vs. 12	6 vs. 12	1 vs. 12	3 vs. 12	1 vs. 12	3 vs. 12
Monotherapy after short term DAPT	Aspirin	Clopidogrel	Aspirin	Aspirin	Aspirin	Aspirin	Aspirin	Ticagrelor	Aspirin	Clopidogrel	Ticagrelor
Stent type	ACS all implanted with E-ZES (Endeavor) and R-ZES (Resolute)	E-ZES (Endeavor)	EES (Xience V)	Biodegradable polymer SES (Tivoli, Essen Tech, Beijing, China)	EES (Xience Prime)	EES (Xience) (34.6%) ZES (Resolute) (33.8%) BES (Biomatrix) (30.4%) Other stents(0.63%)	ZES (Medtronic) (93%) other (7%)	BES (Polymer-based)(94.6%) other stent (6.5%)	Biodegradable polymer SES (COMBO)	EES (Xience)	Bioresorbable polymer SES (Orsiro)
Inclusion	Age 20 years or older; Significant coronary artery stenosis (>50% by visual estimate) which is considered for coronary revascularization with stent implantation	>18 years of age; Clinical indication for PCI; At least one stenosis ≥50% (by visual estimation) Coronary anatomy suitable for percutaneous treatment with E-ZES	Patients aged 18 years or over; eligible for percutaneous coronary intervention (PCI), with at least one Xience V DES implanted	The patient must be ≥18 of age; At least one lesion with a diameter stenosis >70% or more suitable for coronary stent implantation in a vessel with a reference diameter ranging from 2.5 mm to 4.0 mm	Age 20 years old or older; Significant coronary artery stenosis (>50% by visual estimate) considered for coronary revascularization with stent implantation; Stent length ≥28 mm by angiography estimation	Subject must be ≥20 years; Subject must have a culprit lesion in a native coronary artery with significant stenosis (>50% by visual estimate) eligible for stent implantation; Target lesion(s) must be located in a native coronary artery with visually estimated diameter of ≥2.25 mm and ≤4.25 mm	STEMI patients between 18–85 years who underwent primary PCI with DES implantation	Age ≥18 years; Presence of one or more coronary artery stenoses of 50% or more in a native coronary artery or in a saphenous venous or arterial bypass conduit suitable for coronary stent implantation. The vessel should have a reference vessel diameter of at least 2.25 mm	The patient must be ≥18 years of age; Successful COMBO stent implantation (TIMI 3 flow with residual stenosis <20% based visual estimation), with no clinical adverse event during hospitalization [Death, stent thrombosis, stroke, target-vessel revascularization, bleeding (BARC II, III, V)]	Patients received percutaneous coronary intervention with cobalt-chromium everolimus-eluting stent; Patients who are capable of oral dual antiplatelet therapy consisting of asprin and P2Y_12_ receptor antagonist	Patients ≥19 years old; Patients who received new generation sirolimus-eluting (Osiro®) stent implantation for treating ACS
Exclusion	Contraindication to anti-platelet agents and bleeding history within prior 3 months; Cerebral or peripheral atherosclerotic arterial disease, thromboembolic disease or stent thrombosis history; left ventricular ejection fraction <40%; chronic total occlusion; left main disease requiring intervention	STEMI presenting for primary or rescue PCI, PCI with bare metal stent(s) in non-target lesion b6 mo prior to index procedure; Previous treatment with any DES; Scheduled elective surgery within 12 mo post index procedure; Contra-indication, intolerance, or known hypersensibility to aspirin and/or clopidogrel Lesion located in saphenous vein graft; DES in-stent restenosis	Prior DES implantation within 1 year; known platelet level <100,000/μl or known hemorrhagic diathesis; oral anticoagulation therapy or abciximab treatment during hospital stay; contraindications to aspirin or clopidogrel (prasugrel or ticagrelor); major surgery within the preceding 6 weeks; evidence of active gastrointestinal or urogenital bleeding; severe liver failure; any surgery scheduled during the year after enrolment	Patient has a history of bleeding diathesis or coagulopathy or patients in whom anti-platelet and/or anticoagulant therapy is contraindicated or in which patient will not be able to comply with dual antiplatelet therapy for at least 1 year; Left ventricular function <40%; Two or more chronic total occlusions in the proximal half of the epicardial coronary artery which cannot be recannalized	Age >80 years old; Acute ST elevation myocardial infarction within 48 h; Contraindication to anti-platelet agents and bleeding history within prior 3 months; Cerebral vascular accident; Peripheral artery occlusive diseases; Thromboembolic disease; Stent thrombosis; LVEF <40%	Target lesion(s) must be located in a native coronary artery with visually estimated diameter of ≥2.25 mm and ≤4.25 mm; Gastrointestinal or genitourinary bleeding within the prior 3 months, or major surgery within 2 months; An elective surgical procedure is planned that would necessitate interruption of clopidogrel during the first 12 months post enrollment	Known bleeding diathesis or known coagulopathy; History of stent thrombosis; DES in main left coronary artery; Active bleeding, known bleeding diathesis or known coagulopathy; Oral anticoagulant therapy	Planned surgery, including coronary artery bypass graft (CABG) as a staged procedure (hybrid) within 12 months of the index procedure, unless dual antiplatelet therapy is maintained throughout the peri-surgical period; Need for chronic oral anti-coagulation therapy; Active major bleeding or major surgery within the last 30 days	Patients presenting with cardiogenic shock; Patients presenting with cardiogenic shock; Patients requiring permanent DAPT due to comorbidities	Patients requiring oral anticoagulants; Patients with medical history of intracranial hemorrhage; Patients comfirmed to have no tolerability to clopidgorel before enrollment; Patients requiring continuous administration of antiplaelet drugs other than aspirin and P2Y_12_ receptor antagonists at the time of enrollment	Age >80 years; Increased risk of bleeding, anemia, thrombocytopenia; A need for oral anticoagulation therapy; Patients who had history of intracranial hemorrhage
Bleeding	TIMI	GUSTO, BARC	TIMI	BARC	TIMI	BARC	TIMI	BARC	BARC	TIMI	TIMI
Net adverse clinical event, definition	Cardiovascular death, myocardial infarction, stroke, target-vessel revascularization, bleeding	All cause death, myocardial infarction, stroke, major bleeding	All cause death myocardial infarction, stroke, target-vessel revascularization, major bleeding	All-cause death, myocardial infarction, stroke, major bleeding	Cardiac death, myocardial infarction, stroke, major bleeding	all cause death, myocardial infarction, stroke, BARC type 2–5 bleeding	All cause mortality, myocardial infarction, any revascularization, stroke and thrombolysis in myocardial infarction major bleeding	–	all cause mortality, myocardial infarction, stent thrombosis, stroke, target-vessel revascularization, bleeding	cardiovascular death, myocardial infarction, stroke, definite stent thrombosis, and major or minor bleeding	major bleeding, death, myocardial infarction, stent thrombosis, stroke, or target-vessel revascularization
Follow-up (months)	12	12	12	18	12	18	18	24	24	12	12

*RCT, randomized controlled trial; DAPT, dual antiplatelet therapy; ACS, acute coronary syndrome; ZES, zotarolimus-eluting stent; EES, everolimus-eluting stent; BP-SES, biodegradable polymer sirolimus eluting stent; BES, biolimus-eluting stent; TIMI, thrombolysis in myocardial infarction; BARC, bleeding academic research consortium criteria; DES, drug-eluting stent*.

**Table 2:** Baseline characteristics of the patients included.

**Study name**	**RESET**	**OPTIMIZE**	**ITALIC**	**I-LOVE-IT2**	**IVUS-XPL**	**SMART-DATE**	**DAPT-STEMI**	**GLOBAL LEADERS**	**REDUCE**	**STOPDAPT-2**	**TICO**
Patients, *n* (short/standard DAPT)	1,059/1,058	1,563/1,556	912/910	909/920	699/701	1,357/1,355	433/437	7,980/7,988	751/745	1,500/1,509	1,527/1,529
*n*, ACS/Overall	601/2,117	1,000/3,119	792/1,829	1,496/1,829	686/1,400	2,712/2,712	870/870	7,487/15,968	1,496/1,496	1,148/3,009	3,056/3,056
Age (mean)	62.4/62.4	61.3/61.9	61.7/61.5	60.4/60	63/64	62/62.2	59.8/60.2	64.5/64.6	61/60	68.1/69.1	61/61
Men (%)	64.4/62.9	63.5/63.1	80.8/79.2	67.2/68.7	67/70	74.9/75.9	78/76	76.6/76.9	82.6/77.3	78.9/76.5	79/80
Hepertension (%)	62.3/61.4	86.4/88.2	65.2/64.7	61.0/64.8	63/65	49.9/48.7	45/45	74.0/73.3	50.7/50.7	73.7/74.0	50/51
Diabetes (%)	29.8/28.8	35.4/35.3	36.3/37.8	23.2/22.1	36/37	26.9/28.1	13/14	25.7/24.9	21.6/19.5	39/38.0	27/27
Dyslipidemia (%)	57.7/59.9	63.2/63.7	67.1/67.1	25.3/23.4	68/65	24.2/25.2	28/29	69.3/70.0	46.3/44.9	74.4/74.8	61/60
Somker (%)	25.2/22.8	18.6/17.3	50.9/52.7	48.4/49.8	25/24	38.0/40.1	51/47	25.9/26.3	42.1/42.7	26.6/20.6	36/38
Previous MI (%)	1.8/1.6	34.6/34.8	15.6/14.7	17.2/15.8	5/4	2.3/1.7	6/5	23.0/23.6	–	13.8/13.2	4/3
Previous PCI (%)	3.5/3.0	20.9/19.1	24.1/22.5	8.5/6.5	10/10	–	7/4	32.7/32.7	11.7/9.8	33.5/35.1	9/8
Pervious CABG (%)	0.2/0.6	7.1/8.2	6.7/4.9	0.4/0.4	3/2	–	2/0.5	5.6/6.2	2.8/2.8	1.1/2.8	1/1
LVEF	64.2/63.9	–	–	60.8/60.3	62.3/63.1	55.5/55.4	–	–	–	59.8/59.7	–
Multivessel disease (%)	43.1/42.9	–		31.5/31	67/70	43.6/46.6	–	–	36.1/38.8		55/56
**Treated vessels**											
Left main coronary (%)	–	1.2/1.5	1.5/0.9	1.9/1.7	–	2.1/1.3	–	1.9/1.8	–	2.9/2.5	3.0/2.0
Left anterior descending (%)	52.7/53.6	47.9/46.6	73.4/72.3	45.9/45.3	55/56	56.6/61.0	39/43	41.2/42.0	48.0/44.2	55.2/56.6	48/48
Left circumflex (%)	21.0/19.2	23.4/24.3	50.0/47.9	22.9/22.2	20/18	24.4/25.1	21/16	24.3/24.5	–	17.9/20.2	19/19
Right coronary (%)	26.3/27.1	27.6/27.7	53.6/52.1	29.4/30.8	25/26	37.2/36.2	41/41	31.6/30.7	–	29.1/27.2	30/31
Multivessel intervention patients (%)	22.0/23.4	25.34/26.54	49.5/45.7	–	–	19.4/20.7	–	25.5/25.3	–	–	17/18
Total number of stents per patient (*n*)	–	1.6/1.6	1.7/1.7	1.7/1.7	1.6/1.6	1.4/1.5	1.4/1.5	–	–	1.3/1.3	1.37/1.37
Total stent length per patient (mm)	–	19.84/20.01	38.6/37.8	41.0/41.2	46.5/48.2	–	28.5/29.8	–	23.0/23.0	30.3/30.5	35/35
STEMI (%)	–	–	0.1/0.3	13.4/13.7	–	37.5/37.9	100/100	13.3/12.9	49.3/45.2	19.4/17.9	36/36
NSTEMI (%)	–	5.4/5.4	7.3/7.1	11.3/10.7	–	31.5/31.4	–	21.1/21.1	35.6/41.0	5.4/6.6	35/32
UA (%)	40.8/39.9	–	15.7/16.4	58.0/56.5	34/33	31/30.7	–	12.6/12.7	15.2/13.8	12.9/14.2	29/32
Clopidogrel (%)	100/100	100/100	98.9/98.4	100/100	100/100	79.7/81.8	42/42	53/53.2	41.1/40.5	60.2/62.9	36/33
Prasugrel (%)	–	–	1.6/1.8	–	–	–	29/30	–	11.1/9.7	39.6/37.0	0.2/0.26
Ticagrelor (%)	–	–	1/0	–	–	–	29/28	47/47	47.9/49.9	–	73/70

*MI, myocardial infarction; PCI, percutaneous coronary intervention; CABG, coronary artery bypass graft; STEMI, ST-segment elevation myocardial infraction; NSTEMI, Non-ST-segment elevation myocardial infraction; UA, unstable angina*.

### Quality Assessment

The risk of bias for each randomized controlled trial included were presented (Supplementary Figure 1). The random sequence generation methods were introduced in all 11 trials, including computer generated random or interactive web-based response systems. The central random assignment was also used in all trials. Although all trials were open-label studies, lack of blinding was considered to unlikely affect our outcome. In addition, 10 trials clinical data were collected and analyzed by an independent committee in each trial, and the risk of detection bias was low. No independent adjudication of clinical events was implemented in one trial (20). The reasons for the missing data were found in all trials, which were described in each trial and analyzed by intention-to-treat analysis. All trials were registered at clinicaltrials.gov and certified by the national clinical trial. One trial was terminated prematurely due to recruitment problems (17).

The quality assessments of each endpoint evidence were shown (Supplementary Table 1). The evidence quality of endpoint was determined to be high for net adverse clinical events, and was moderate for myocardial infarction, definite or probable stent thrombosis, major bleeding, all-cause death, cardiovascular death, stroke and target vessel revascularization.

The trial sequential analysis showed that the cumulative Z curve crossed the conventional boundary, but it did not cross the trial sequential monitoring boundary and required information size, which suggested that although the meta-analysis had concluded that the short-term DAPT might decrease the major bleeding complications, more trials are needed to control type I error (Supplementary Figure 2). This meta-analysis showed that there was no significant difference between short-term DAPT and standard DAPT in terms of myocardial infarction, definite or probable stent thrombosis, all-cause death, cardiovascular death, stroke, target vessel revascularization and net adverse clinical events, and the trial sequential analysis indicated that more trials are needed to control random errors (Supplementary Figure 3).

### The Primary Endpoints

#### Myocardial Infarction, Definite or Probable Stent Thrombosis, and Major Bleeding

**Figure 2:**
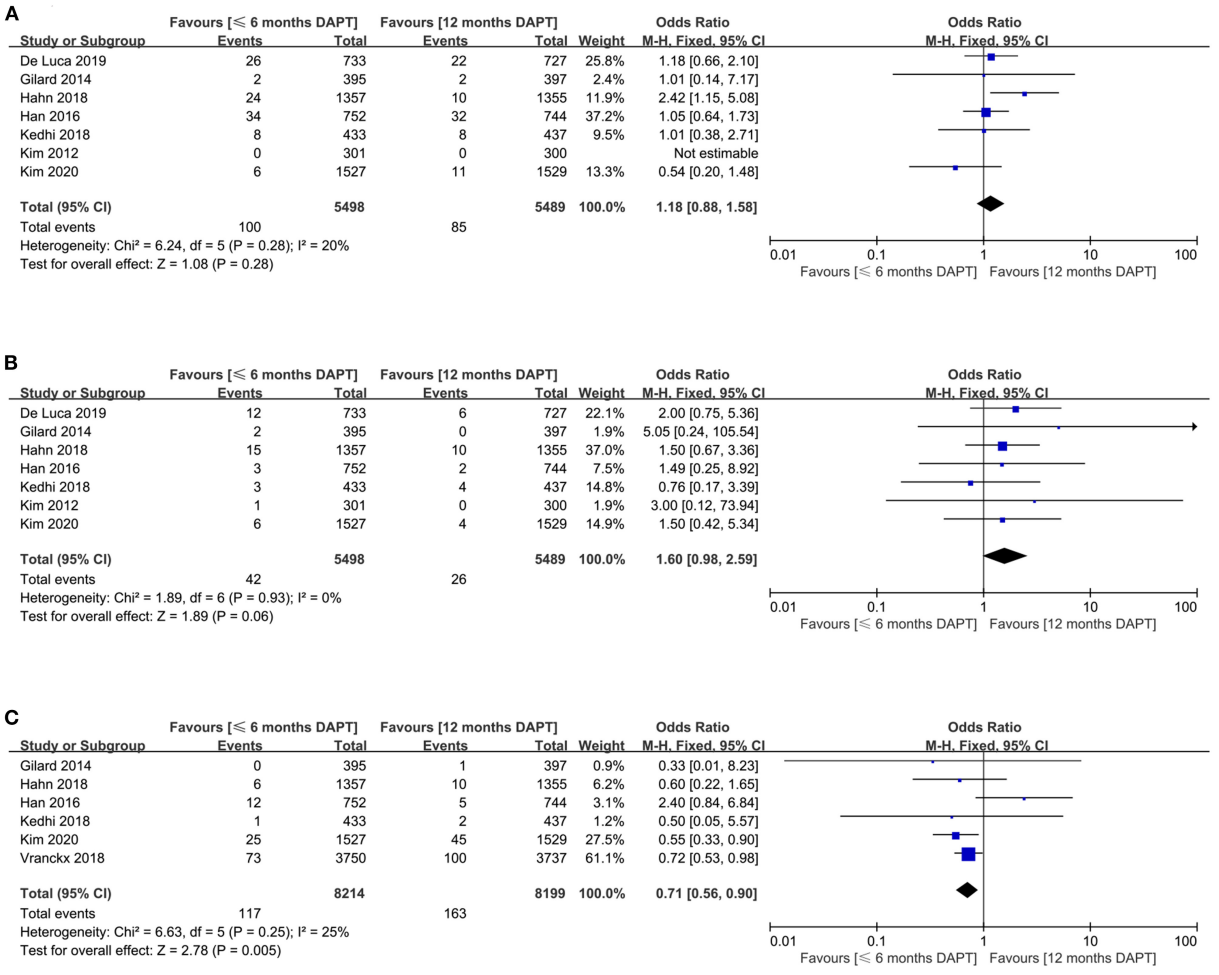
Comparison of primary endpoints between short-term and standard DAPT groups. **(A)** myocardial infarction, **(B)** definite or probable stent thrombosis, **(C)** major bleeding.

The myocardial infarction was reported in seven studies (10,987 patients). The risk of myocardial infarction was similar in patients received ≤6 months DAPT compared with 12 months DAPT (1.8 vs. 1.5%, OR 1.18, 0.88–1.58, *P* = 0.28, *I*^2^ = 20%, *P*_*Heterogeneity*_ = 0.28) (Figure 2A). For this result, no asymmetry was identified in the funnel plot by visual estimation (Supplementary Figure 4), and Egger's and Begg's tests were *P* = 0.764 and *P* = 1, respectively.

The definite or probable stent thrombosis was extracted from seven trials with a total of 10,987 patients. There was no significant difference in definite or probable stent thrombosis between patients received ≤ 6months DAPT compared with 12 months DAPT (0.8 vs. 0.5%, OR 1.60, 0.98–2.59, *P* = 0.06, *I*^2^ = 0%, *P*_*Heterogeneity*_ = 0.93) (Figure 2B). The funnel plot showed slight asymmetry by visual estimation (Supplementary Figure 4). However, Egger's and Begg's tests did not confirm publication bias (*P* = 0.529 and *P* = 0.368, respectively).

The meta-analysis involving 16,413 patients in six studies evaluated the major bleeding outcomes. The shorter DAPT strategy could significantly reduce the risk of major bleeding compared with the standard DAPT strategy (1.4 vs. 2%, OR 0.71, 0.56–0.90, *P* = 0.005, *I*^2^ = 25%) (Figure 2C). According to visual estimation, the funnel plot was slightly asymmetrical (Supplementary Figure 4). However, no publication bias was found in Egger's and Begg's tests (*P* = 0.867 and *P* = 1, respectively).

### The Secondary Endpoints

#### All-Cause Death and Cardiovascular Death

**Figure 3:**
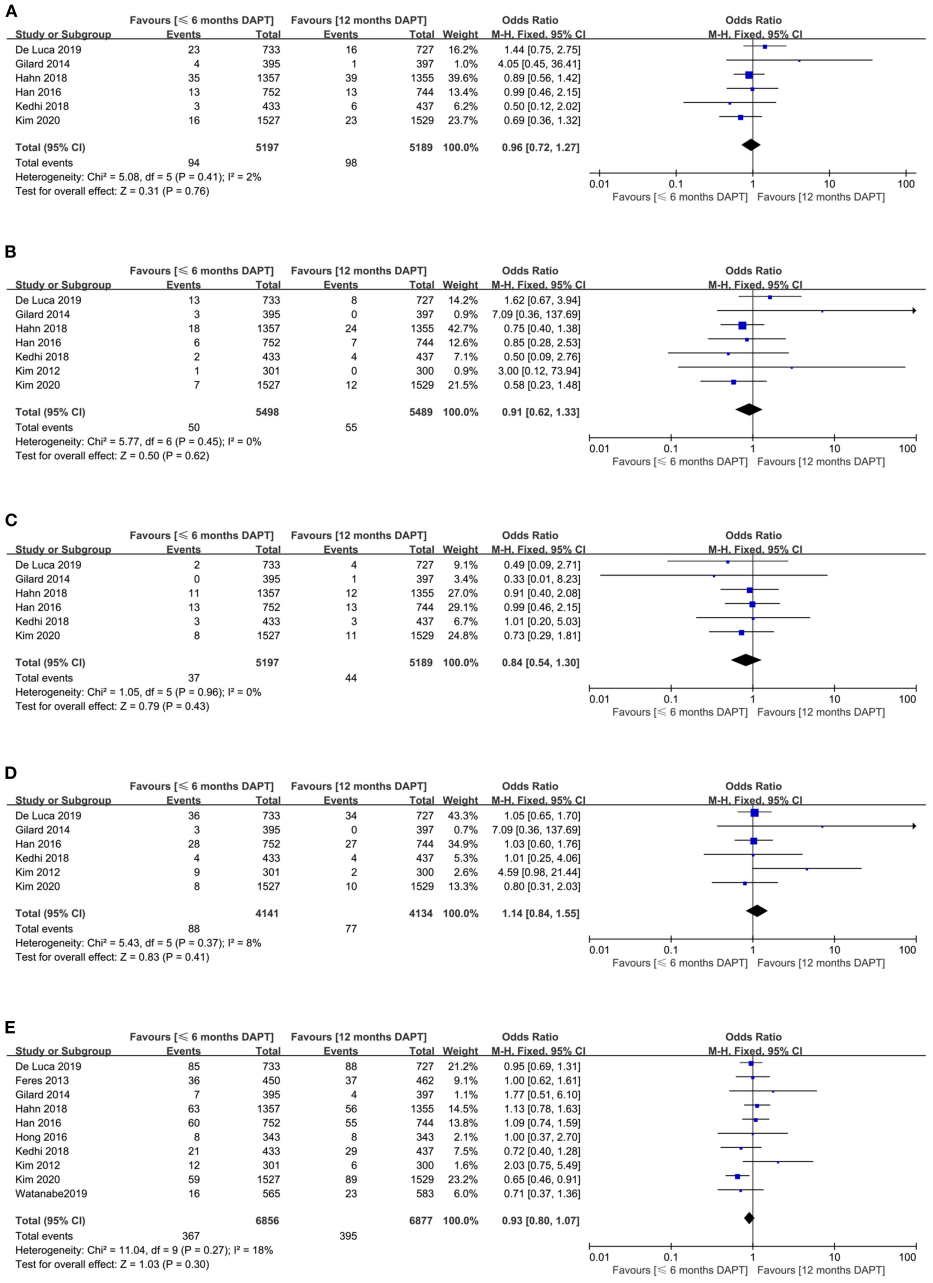
Comparison of secondary endpoints between short-term and standard DAPT groups. **(A)** all-cause death, **(B)** cardiovascular death, **(C)** stroke, **(D)** target vessel revascularization, **(E)** net adverse clinical events.

The all-cause death appeared in six studies (10,386 patients). There was no significant difference in all-cause death between the two strategies (1.8 vs. 1.9%, OR 0.96, 0.72–1.27, *P* = 0.76, *I*^2^ = 2%, *P*_*Heterogeneity*_ = 0.41) (Figure 3A). Meanwhile, funnel plot by visual inspection, and Egger's and Begg's tests did not detect significant publication bias (*P* = 0.631, and *P* = 0.707, respectively) (Supplementary Figure 4).

The cardiovascular death was reported in seven studies (10,987 patients). They showed that the incidence of cardiovascular death was similar between the two groups (0.9 vs. 1%, OR 0.91, 0.62–1.33, *P* = 0.62, *I*^2^ = 0%, *P*_*Heterogeneity*_ = 0.45) (Figure 3B). The funnel plot revealed slight asymmetry according to visual estimation (Supplementary Figure 4). However, the publication bias has not been confirmed by Egger's and Begg's tests (*P* = 0.332 and *P* =0.707, respectively).

#### Stroke and Target Vessel Revascularization

The stroke as the secondary endpoint was presented in six studies (10,386 patients). There was no significant difference in stroke endpoint between the two strategies (0.7 vs. 0.8%, OR 0.84, 0.54–1.30, *P* = 0.43, *I*^2^ = 0%, *P*_*Heterogeneity*_ = 0.96) (Figure 3C). Although the funnel plot was slightly asymmetric (Supplementary Figure 4), Egger's and Begg's tests did not confirm publication bias (*P* = 0.122 and *P* = 0.06, respectively).

Six studies with a total of 8,275 patients reported the target vessel revascularization. The study manifested that there was no significant difference in target vessel revascularization between the two strategies (2.1 vs. 1.9%, OR 1.14, 0.84–1.55, *P* = 0.41, *I*^2^ = 8%, *P*_*Heterogeneity*_ = 0.37) (Figure 3D). The funnel plot was slightly asymmetrical (Supplementary Figure 4), while Egger's and Begg's tests did not show publication bias (*P* = 0.152 and *P* = 0.26, respectively).

#### Net Adverse Clinical Events

Ten studies with a total of 13,733 patients provided data on the second endpoint of net adverse clinical events. There was no significant difference in the incidence of net adverse clinical events between the two strategies (5.4 vs. 5.7%, OR 0.93, 0.80–1.07, *P* = 0.3, *I*^2^ = 18%, *P*_*Heterogeneity*_ = 0.27) (Figure 3E). The funnel plot exhibited symmetry (Supplementary Figure 4), and there was no publication bias in Egger's and Begg's tests (*P* = 0.368 and *P* = 0.283, respectively).

### Subgroup Analysis

In most subgroup, there was no significant interactions between therapeutic effect and various research characteristics. A treatment-by-subgroup interaction was noted for P2Y_12_ inhibitor in major bleeding. The results of subgroup analysis were shown (Supplementary Figures 5, 6). Similar results were also observed when comparing myocardial infarction, definite or probable stent thrombosis, all-cause death, cardiovascular death, stroke, target vessel revascularization, and net adverse clinical events in different subgroups (Supplementary Figures 5, 6). The risk of major bleeding in patients taking ticagrelor alone after short-term DAPT was significantly lower (OR 0.67, 0.51–0.86, *P* = 0.002) than that of patients taking aspirin alone (OR 1.05, 0.55–1.99, *P* = 0.881) (Supplementary Figure 5C). The incidence of major bleeding in patients with short-term DAPT for 1 or 3 months (OR 0.72, 0.53–0.98, *P* = 0.036; OR 0.54, 0.33–0.89, *P* = 0.015) was significantly lower than that of patients with short-term DAPT for 6 months (OR 1.05, 0.55–1.99, *P* = 0.881) (Supplementary Figure 5C). Patients implanted with third-generation of DES had significantly lower incidence of major bleeding (OR 0.72, 0.56–0.93, *P* = 0.01) (Supplementary Figure 5C), and lower risk of target vessel revascularization (OR 1.01, 0.72–1.41, *P* = 0.971) (Supplementary Figure 6D) than patients implanted with second-generation of DES in shorter duration of DAPT.

## Discussion

The current findings indicate that 1–6 months DAPT significantly reduced major bleeding complications in patients with acute coronary syndrome receiving a new generation stents without increasing the risk of ischemic events and death, compared with standard 12 months DAPT.

In the antiplatelet therapy, it is important to balance the risk of ischemic and bleeding complications, both of which are closely related to adverse outcomes following PCI. Although a previous study has shown that the incidence of ischemia and bleeding is similar between patients treated with DAPT for ≤6 months vs. those treated with DAPT for 12 months (22). However, this meta-analysis showed that the standard DAPT was associated with a higher risk of major bleeding in patients with acute coronary syndrome compared with the short-term DAPT, and the discrimination was even more pronounced when the study was limited to enroll only patients with acute coronary syndrome, including a novel P2Y_12_ inhibitor, monotherapy after short-term DAPT with ticagrelor, and short-term DAPT for ≤3 months. Shortening the duration of DAPT could reduce the risk of major bleeding in this analysis, while the lack of difference in that analysis may be due to the different sample sizes. A study has found that the major bleeding was closely related to mortality after PCI, and the total mortality could be reduced by reducing the risk of bleeding (23). In this regard, patients with DAPT can benefit from shortening the duration.

Patients with acute coronary syndrome are different from those with stable coronary artery disease, and who carry a higher risk of recurrent ischemic events. Therefore, shortening the duration of DAPT therapy may increase the risk of ischemic events for acute coronary syndrome patients underwent PCI. Implementation of new generation of DES, and the widespread use of a novel generation of P2Y_12_ inhibitors have resulted in a reduction of definite or probable stent thrombosis or myocardial infarction in acute coronary syndrome patients with PCI (4, 24). Although the difference in myocardial infarction and definite or probable stent thrombosis was not statistically significance in this meta-analysis, a trend toward increased risk of myocardial infarction (1.8 vs. 1.5%, OR 1.18, 0.88–1.58, *P* = 0.28) and definite or probable stent thrombosis (0.8 vs. 0.5%, OR 1.60, 0.98–2.59, *P* = 0.06) was observed in the short-term DAPT strategy, which be worth special attention. The SMART-DADE study showed that the short-term DAPT strategy significantly increased the myocardial infarction, which might be due to the difference in sample size, the risk of recurrent ischemic events for patients enrolled (9). In conclusion, due to the persistent bleeding risk with prolonged DAPT, clinicians should consider shortening the duration of DAPT, combined with effective medical management and adjustment of risk factors, to prevent definite or probable stent thrombosis and myocardial infarction.

Considering the new generation of DES, the bioresorbable polymer sirolimus-eluting stents based thin strut are expected to reduce the incidence of thrombosis and vascular injury, as well as accelerate endothelialization to obtain the best clinical outcomes (4, 25). Although all-cause death, cardiovascular death, stroke, target vessel revascularization, and net adverse clinical events presented similar risks between short-term and standard DAPT groups in this meta-analysis. The finding of the DAPT trial was an increased risk of mortality in patients treated with prolonged DAPT, which was attributed to increased non-cardiovascular mortality due to cancer, bleeding, and trauma related deaths (26). Of note, although stent thrombosis and myocardial infarction can be reduced by using this strategy, the all-cause mortality increases with the prolongation of DAPT. However, this reduction did not result in a decrease in cardiac mortality with longer DAPT. The number of deaths due to early diagnosis and treatment of myocardial infarction was lower than that in the previous decade (27). Thus, with the extension of DAPT, the non-cardiac mortality was not offset by any benefit of reducing cardiac mortality, resulting in a higher all-cause mortality. From this point of view, the future development direction requires more efforts to accurately determine the beneficiaries of shortening DAPT.

Overall, the results of present meta-analysis seem to offer new opportunities for shortening DAPT duration, even in the context of acute coronary syndrome. The short-term DAPT followed by P2Y_12_ inhibitor or aspirin alone is an alternative, and the best P2Y_12_ inhibitor remains to be established. The subgroup analysis showed that patients taking ticagrelor alone after DAPT had a significantly lower risk of major bleeding compared with aspirin alone. This may be because the duration of DAPT in studies with ticagrelor monotherapy was shorter than that in studies with aspirin monotherapy, such as GLOBAL LEADERS study of 1 month DAPT and TICO study of 3 months DAPT. Ticagrelor alone after DAPT was associated with a lower risk of definite or probable stent thrombosis, which may be due to its stronger inhibition of platelet aggregation than aspirin. In addition, if a patient is treated with a P2Y_12_ inhibitors as monotherapy after PCI, it is unclear whether this strategy should be continued indefinitely beyond 12 months. In this meta-analysis, patients with the new generation of stents included the second-and third-generation of DES in this meta-analysis. The subgroup analysis showed that compared with the second generation of DES, the incidence of major bleeding and target vessel revascularization was significantly reduced in patients with third-generation of DES in shorter duration of DAPT. This may be due to the fact that the second-generation of DES used polymers embedded in the vessels walls permanently, while third-generation of DES used biodegradable polymers, which can reduce persistent inflammatory and allergic reactions in the vessels.

### Limitation

As with any meta-analysis, this study has the limitations of the original research. Firstly, the definitions of some clinical endpoints vary slightly in different trials, and may potentially reduce accuracy. However, no significant heterogeneity was found in our primary and secondary endpoints. Secondly, more than half of the studies included were subgroup analyses of large randomized controlled trials, including patients with stable angina pectoris, which may have contributed to bias. Thirdly, patients with acute coronary syndrome in this meta-analysis was only partially representative, as some of the original study excluded higher risk patients (such as age > 80 years old, history of hemorrhage, a need for oral anticoagulation therapy, DES in main left coronary artery, history of diabetes or renal failure) and neither we could consider patients with left main disease or cardiogenic shock, which were excluded in the majority of trial. Fourthly, in this meta-analysis, 63.3% of patients took clopidogrel as an adjunct to aspirin, and which may not apply to ticagrelor or prasugrel. Finally, most of studies included in the meta-analysis were not masked, although the impact on the endpoint was small or not.

### Conclusion

The shorter 1–6 months DAPT significantly reduce major bleeding complications without increasing the risk of myocardial infarction, definite or probable stent thrombosis, all-cause death, cardiac death, stroke, target vessel revascularization and net adverse clinical events compared with the standard 12 months DAPT. Despite shortening DAPT might be reasonable for some patients, individualized approach should still be preferred in absence of data from larger trials. Future research should focus on designing and testing algorithms that can identify patients who benefit from shortening DAPT by balancing the risk of ischemic and bleeding.

## Data Availability Statement

The original contributions presented in the study are included in the article/Supplementary Material, further inquiries can be directed to the corresponding author/s.

## Author Contributions

W-JZ: study design, data collection, data analysis, and manuscript. XQ: data collection, data analysis, and validation. W-FG, X-YL, and YL: data collection and validation. Z-LW: scientific revision of the manuscript. All authors contributed to the article and approved the submitted version.

## Conflict of Interest

The authors declare that the research was conducted in the absence of any commercial or financial relationships that could be construed as a potential conflict of interest.
