# Supplementary material for: Duration of Dual Antiplatelet Therapy in Patients With Acute Coronary Syndrome Treated With New Generation Stents: A Meta-Analysis of Randomized Controlled Trials
Source: Front Cardiovasc Med. 2021 Feb 3;8:615396. doi: 10.3389/fcvm.2021.615396 (PMC7886789; doi:10.3389/fcvm.2021.615396)
Supplement: Supplementary file 1 [file Data_Sheet_1.docx]

**Supplemental Table 1.** Quality assessments of GRADE evidence quality for each endpoint.


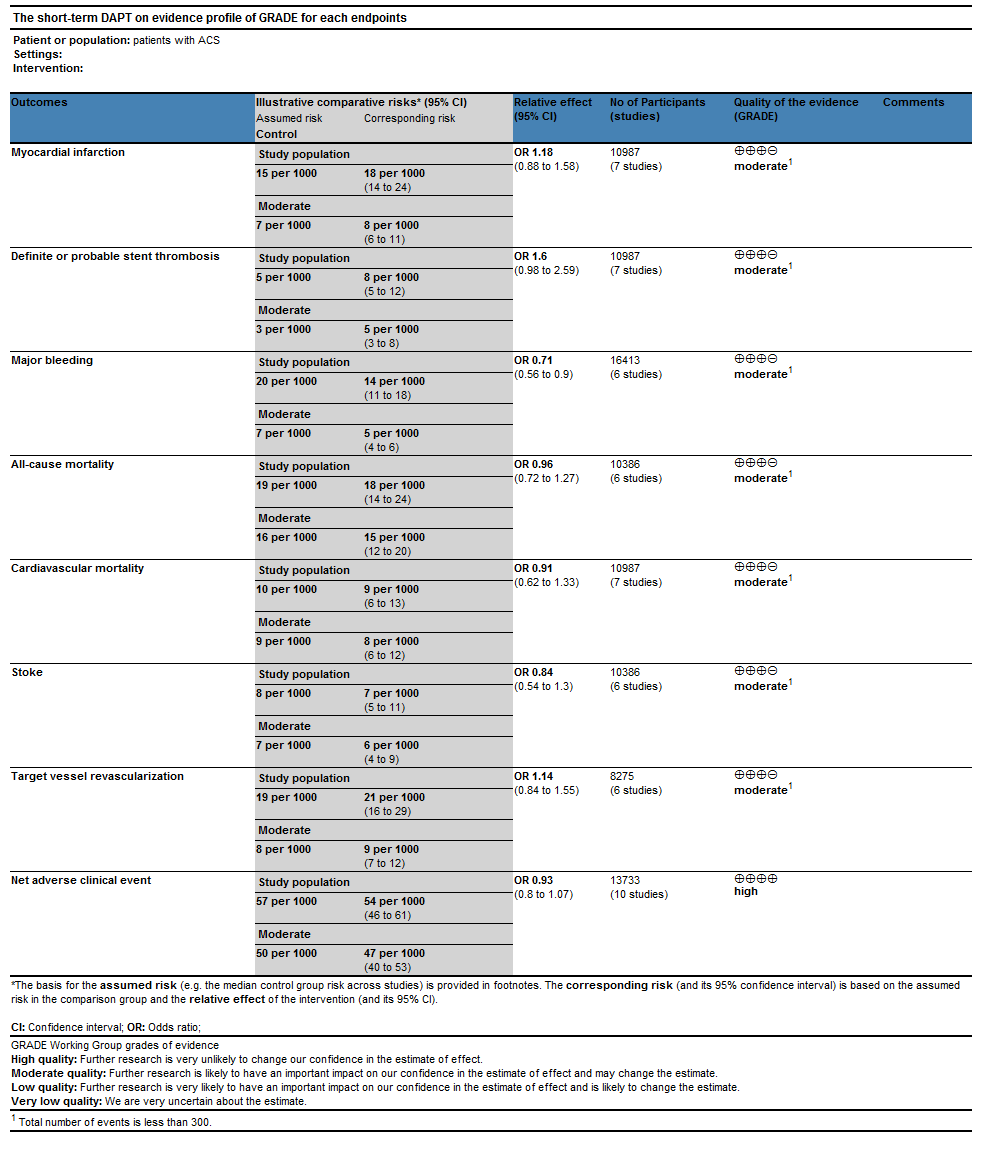


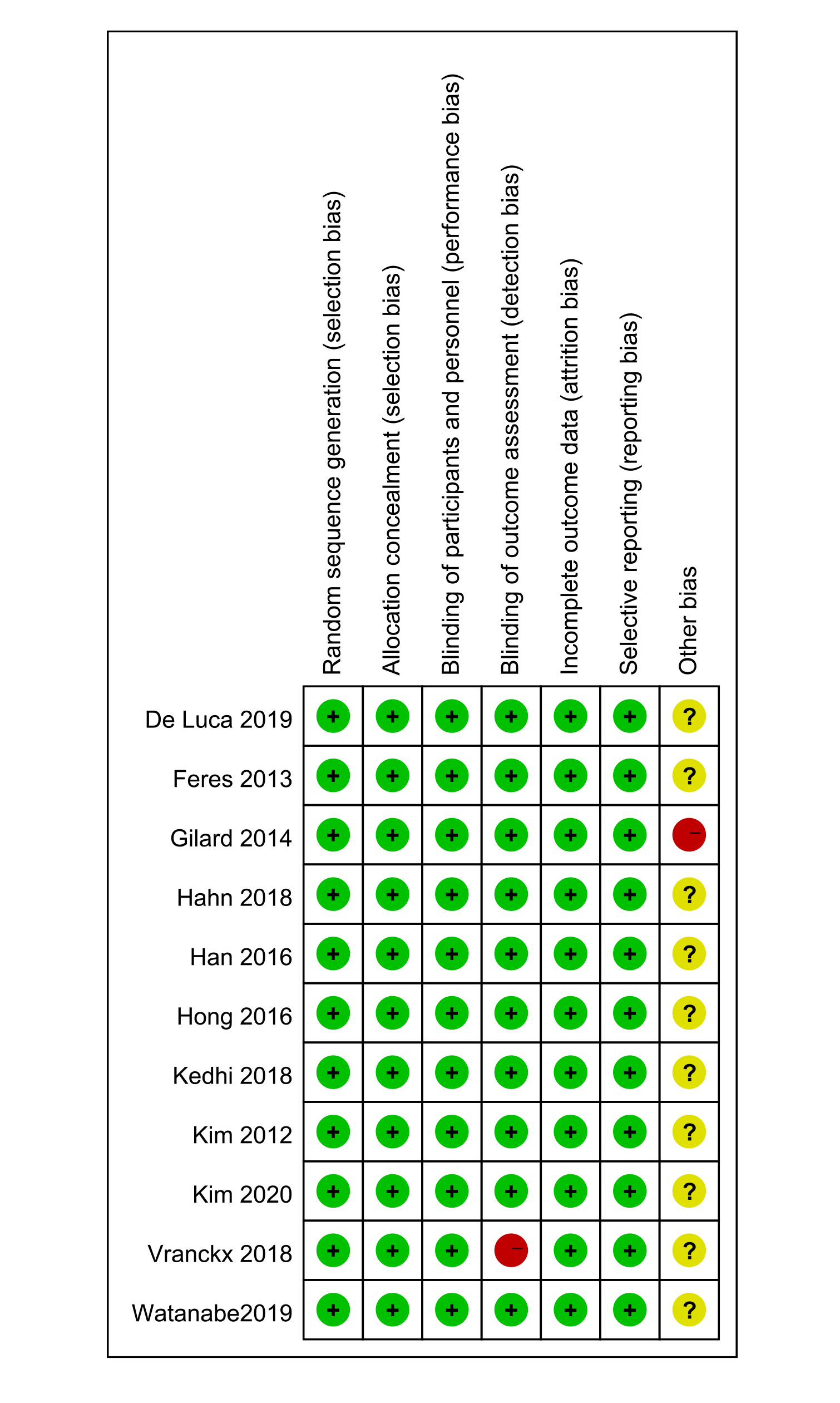


**Supplemental** **Figure 1.** Assessing risk of bias for each randomized controlled trial included.

**
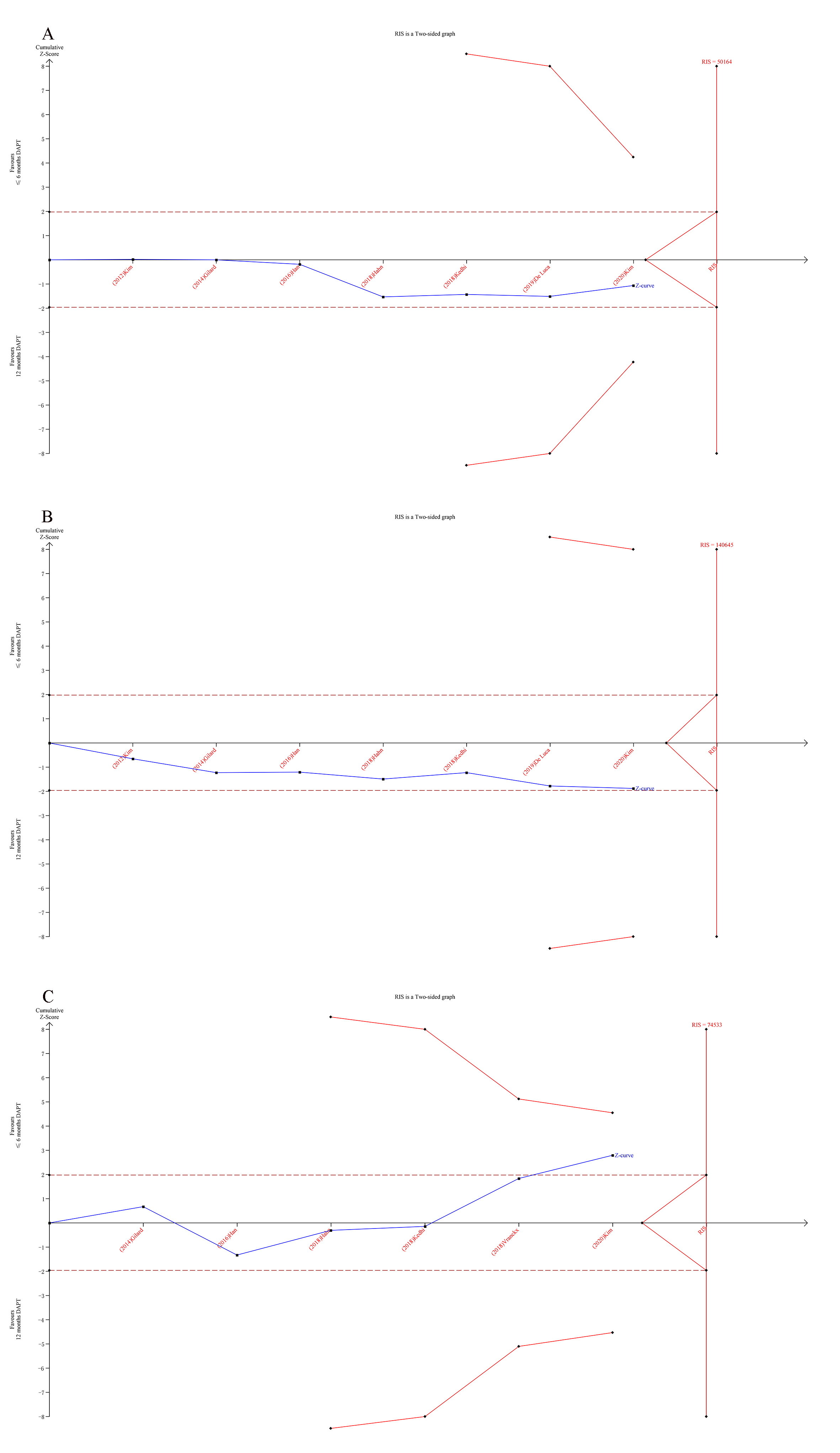
**

**Supplemental Figure 2.** Trial Sequential Analysis for primary endpoints of (A) myocardial infarction, (B) definite or probable stent thrombosis, (C) major bleeding.

**
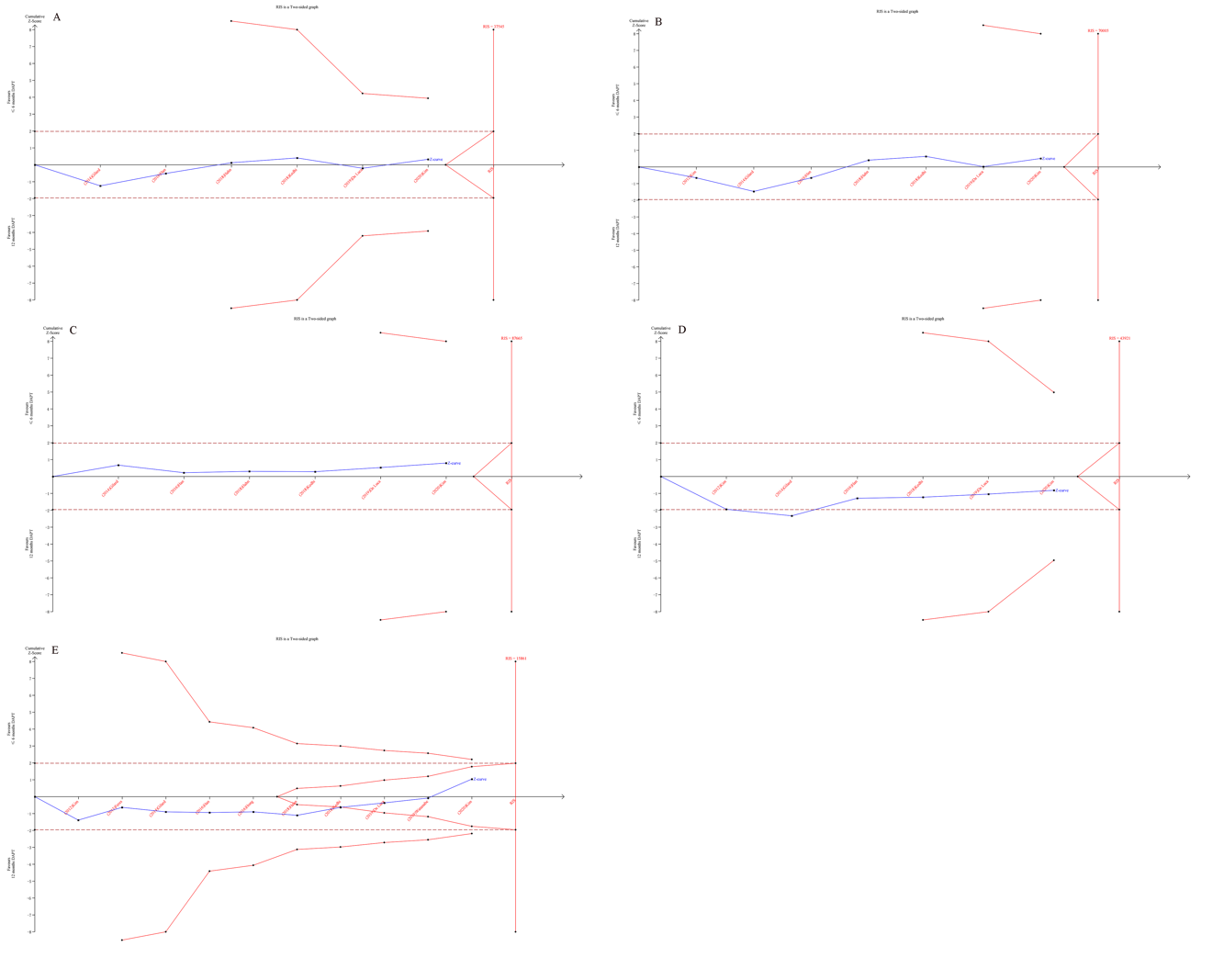
**

**Supplemental Figure 3.** Trial Sequential Analysis for secondary endpoints of (A) all-cause death, (B) cardiovascular death, (C) stroke, (D) target vessel revascularization, (E) net adverse clinical events.


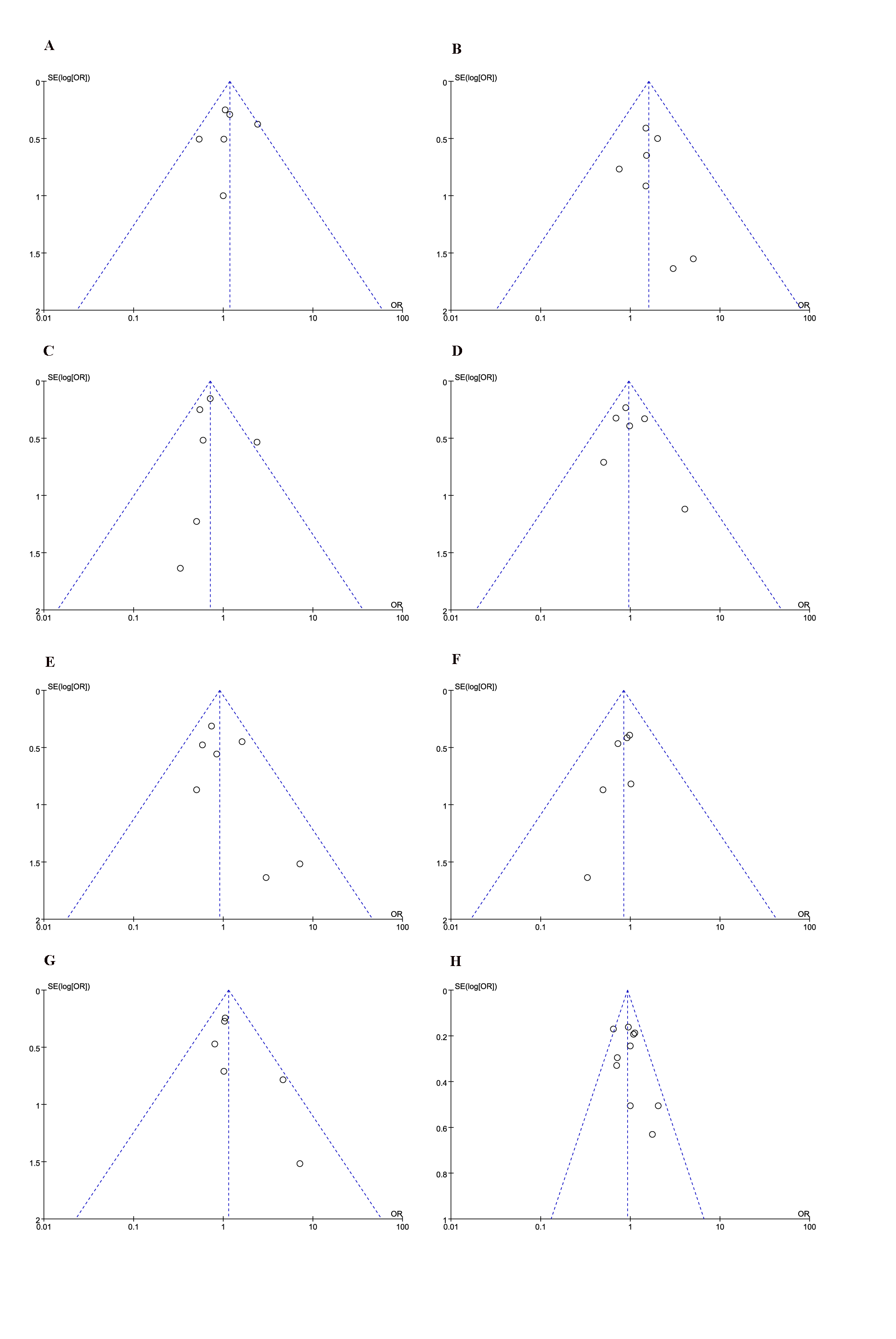


**Supplemental Figure 4.** Funnel plot for each endpoint of (A) myocardial infarction, (B) definite or probable stent thrombosis, (C) major bleeding, (D) all-cause death, (E) cardiovascular death, (F) stroke, (G) target vessel revascularization, (H) net adverse clinical events.

**
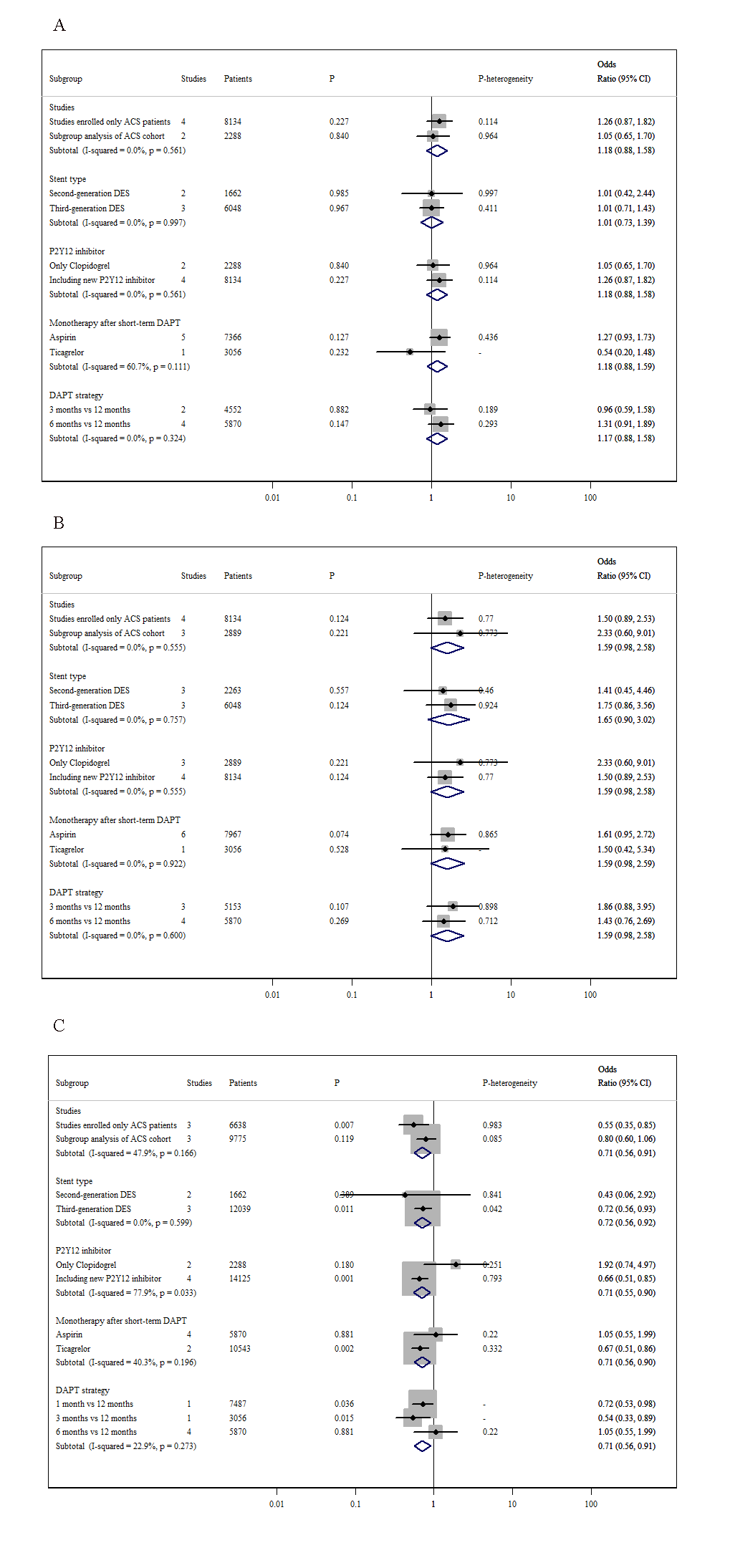
**

**Supplemental Figure 5.** Subgroup analysis for primary endpoints of (A) myocardial infarction, (B) definite or probable stent thrombosis, (C) major bleeding.


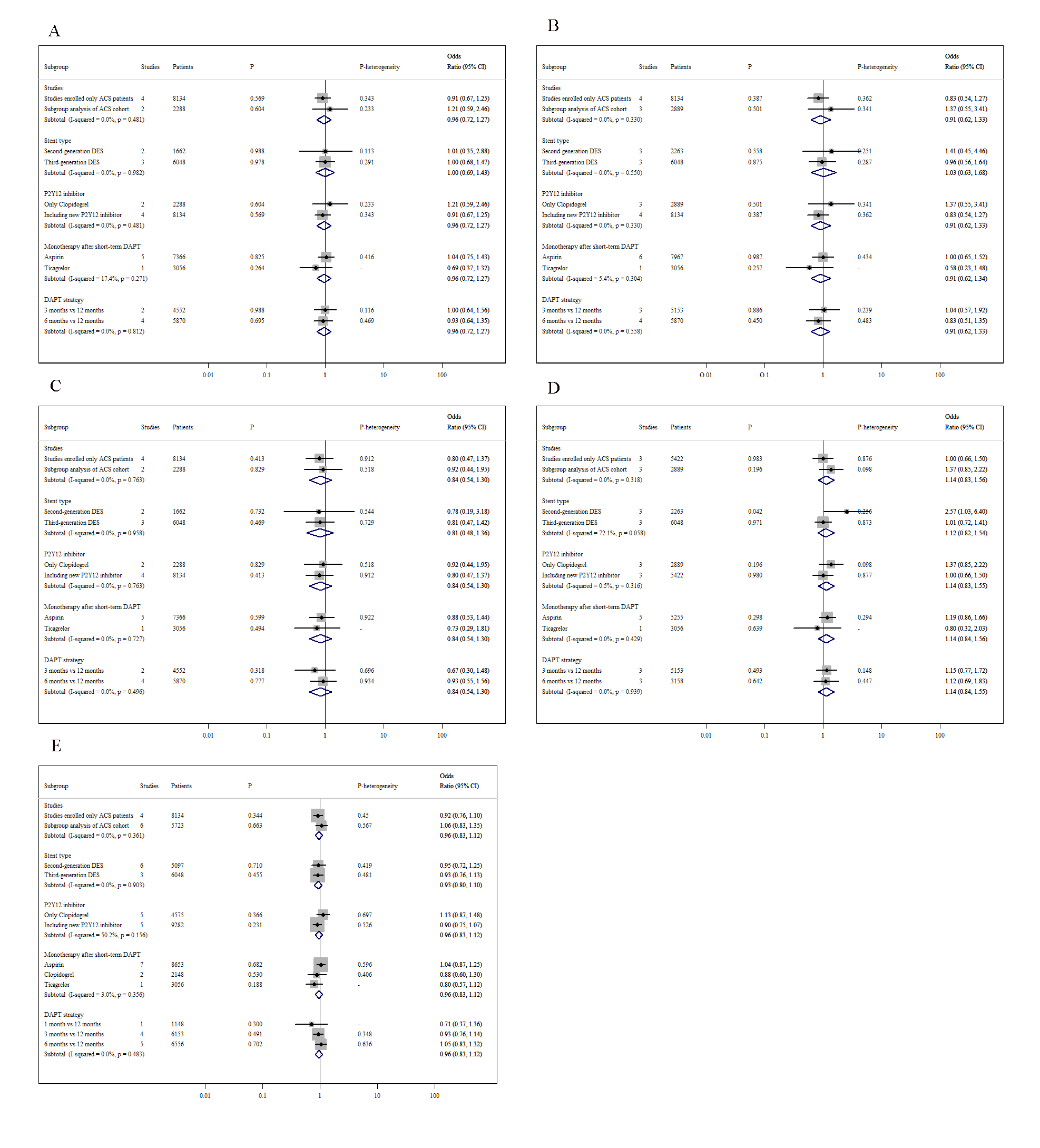


**Supplemental Figure 6.** Subgroup analysis for secondary endpoints of (A) all-cause death, (B) cardiovascular death, (C) stroke, (D) target vessel revascularization, (E) net adverse clinical events.
